# Adenovirus-mediated delivery of herpes simplex virus thymidine kinase administration improves outcome of recurrent high-grade glioma

**DOI:** 10.18632/oncotarget.6737

**Published:** 2015-12-23

**Authors:** Nan Ji, Danhui Weng, Cang Liu, Zheng Gu, Shizhang Chen, Ying Guo, Zhong Fan, Xiao Wang, Jianfei Chen, Yanyan Zhao, Jianfeng Zhou, Jisheng Wang, Ding Ma, Ning Li

**Affiliations:** ^1^ Department of Neurosurgery, Beijing Tiantan Hospital, Capital Medical University, Beijing, P.R. China; ^2^ Beijing YouAn Hospital, Capital Medical University, Beijing, P.R. China; ^3^ Tongji Hospital Affiliated with Tongji Medical College of Huazhong University of Science and Technology, Wuhan, P.R. China; ^4^ Beijing Friendship Hospital, Capital Medical University, Beijing, P.R. China; ^5^ Beijing Chao-Yang Hospital, Capital Medical University, Beijing, P.R. China; ^6^ Beijing Key Laboratory of Brian Tumor, Beijing, P.R. China

**Keywords:** recurrent high-grade glioma, gene therapy, ADV-TK, glioblastoma

## Abstract

**Background:**

This randomized, open-label, multicenter, phase II clinical trial was conducted to assess the anti-tumor efficacy and safety of replication-deficient adenovirus mutant thymidine kinase (ADV-TK) in combination with ganciclovir administration in patients with recurrent high-grade glioma (HGG).

**Patients and Methods:**

53 patients with recurrent HGG were randomly allocated to receive intra-arterial cerebral infusion of ADV-TK or conventional treatments. The primary end point was 6-month progression-free survival (PFS-6). Secondary end points included progression-free survival (PFS), overall survival (OS), safety, and clinical benefit. This trial is registered with Clinicaltrials.gov, NCT00870181.

**Results:**

In ADV-TK group, PFS-6 was 54.5%, the median PFS was 29.6 weeks, the median OS was 45.4 weeks, and better survivals were achieved when compared with control group. The one-year PFS and OS were 22.7% and 44.6% in ADV-TK group respectively, and clinical benefit was 68.2%. There are 2 patients alive for more than 4 years without progression in ADV-TK group. In the subgroup of glioblastoma received ADV-TK, PFS-6 was 71.4%, median PFS was 34.9 weeks, median OS was 45.7 weeks respectively, much better than those in control group. The one-year PFS and OS were 35.7% and 50.0% in ADV-TK group respectively. ADV-TK/ganciclovir gene therapy was well tolerated, and no treatment-related severe adverse events were noted.

**Conclusion:**

Our study demonstrated a notable improvement of PFS-6, PFS and OS in ADV-TK treated group, and the efficacy and safety appear to be comparable to other reported treatments used for recurrent HGG. ADV-TK gene therapy is therefore a valuable therapeutic option for recurrent HGG.

## INTRODUCTION

Treatment options for patients with recurrent GBMs and AAs remain limited. The treatments include resection with or without carmustine (bis-chloroethylnitrosourea, BCNU) wafer placement in selected patients with local recurrence, radiotherapy, chemotherapy, the anti-angiogenic agent bevacizumab alone or in combination with chemotherapy, or alternating electric field therapy [1]. Surgery prolongs survival only to a limited degree, and the benefits of repeated radiotherapy are unclear. The repeated administration of TMZ with the same schedule of the initial treatment has not generally been recommended in recurrent patients due to the risk of cumulative toxicity and to the possibility of chemotherapy resistance. Development of alternative treatment modalities, preferably with mechanisms of action dissimilar to those of standard therapies, is therefore urgently needed.

Most brain tumors are localized lesions of rapidly dividing cells in a background of non-dividing neurons. They rarely metastasize outside of the central nervous system, and recurrence occurs usually at the site of original lesion, making them highly amenable to gene therapy. Thus, several gene-therapy approaches have been studied in glioma. The first clinical gene therapy trials against brain cancer were registered in 1992, using the *ex-vivo* modification of autologous tumor cells with a retrovirus to express the gene encodinginterleukin-2 in relapsed/refractory neuroblastoma [2]. Later, herpes simplex virus (HSV) thymidine kinase (TK) suicide gene therapy using retrovirus-producing packaging cells, followed by intravenous ganciclovir therapy was reported to be used in primary [3] and recurrent [4, 5] rain-cancer patients. Two phase I studies demonstrated the safety and efficacy of replication-deficient adenovirus HSV-TK/ganciclovir gene therapy by comparing with replication-deficient retrovirus therapy or retrovirus-producing packaging cells therapy [6, 7]. In 2004, Immonen et al. reported the first randomized, controlled clinical trial with non-replicable adenovirus HSV-TK/ganciclovir (AdHSV-TK/ganciclovir) gene therapy. AdHSV-TK/ganciclovir gene therapy increased the median survival time and was well tolerated without significant safety issues [8].

Building on these promising results, an international, open-label, randomized, parallel group multicenter phase III clinical trial using the first-generation replication-deficient adenovirus containing the cDNA for HSV-TK (sitimagenecer adenovec; Cerepro^®^, Ark Therapeutics Ltd., London, UK) for newly diagnosed operable HGG patients was reported recently [9]. Their results showed that additional Cerepro^®^ therapy prolonged median time to death or re-intervention of the patients, there was no difference between groups in terms of overall survival (OS), and patients received Cerepro^®^ suffered more treatment-related adverse events, such as hemiparesis and aphasia. Although this study made a step forward from the earlier retrovirus-based phase III study, the limitation of the efficacy of Cerepro^®^ may due to the transduction efficiency.

In 2007, our research group reported the encouraging clinical results of ADV-TK/ganciclovir therapy in advanced hepatocellular carcinoma (HCC). This phase II clinical trial revealed increased survival and safety in the non-vascular invasion HCC patients who received liver transplantation and ADV-TK/ganciclovir adjuvant gene therapy; the 3-year overall survival (OS) rate was 100% and the recurrence-free survival (RFS) rate was 83.3% [10]. In 2009, we reported a phase I clinical trial with ADV-TK/GCV therapy in 18 patients with head and neck cancer and nasopharyngeal carcinoma (NPC), with the aim to determine the safety profile, humoral immune response and biologic activity of a single intra-tumor injection of ADV-TK followed by GCV therapeutic approach [11]. Our findings suggested that ADV-TK gene therapy can be administered safely to cancer patients, and achieved a local response with few environmental effects.

Intra-arterial chemotherapy is a strategy of dose intensification that results in more concentrated and localized delivery of chemotherapy to brain tumors. Since ADV-TK showed good tolerance and safety, we designed a randomized, open-label, multicenter, phase II clinical trial to assess the anti-tumor efficacy and safety of intra-arterial cerebral infusion of ADV-TK in combination with systemic intravenous ganciclovir in patients with recurrent malignant glioma.

## RESULTS

### Patient characteristics and treatment

**Figure 1:**
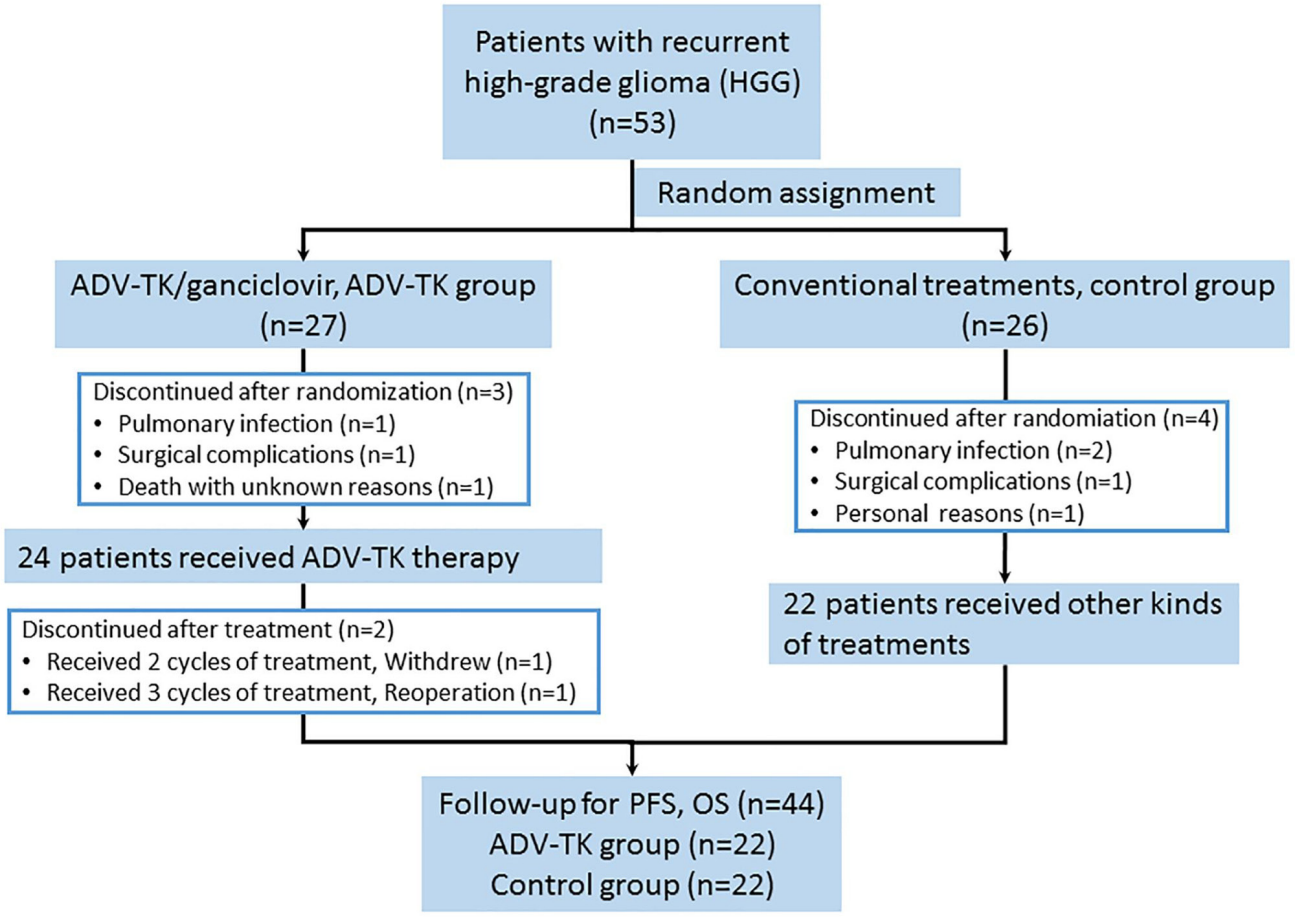
CONSORT diagram

A total of 53 patients were enrolled from three centers in randomization, 27 patients allocated in ADV-TK group and 26 patients allocated in the control group. There were 7 patients, 3 in ADV-TK group and 4 in control group, withdrew after randomization for refusing to continue the trial. In ADV-TK group, one patient was lost to follow-up after two cycles of gene therapy, and one patient refused to continue medical treatment after completing three cycles of treatment. Finally, 44 patients were therefore included in the PFS, OS, and safety analyses, 22 patients in ADV-TK group and 22 patients in control group (CONSORT diagram, Figure 1). Demographic data are listed in Table 1. Baseline characteristics were similar between the two groups.

**Table 1:** Patient demographic and clinical characteristics

Characteristic	ADV-TK group (n=22)	Control group (n=22)	*P*
**Sex**			0.314
**Male**	18	15	
**Female**	4	7	
**Age (yr)**			0.86
**Median**	49	54	
**Range**	28–71	20–69	
**WHO tumor grade**			0.314
**Grade 3**	8	4	
**Grade 4**	14	18	
**Histology**			
**AA/AO/AG/OTHERS**	8	4	0.108
**Glioblastoma**	14	18	
**Time interval before randomization (Week)**			0.387
**Median/Mean**	2.7/6.3	3.4/7.6	
**Range**	0-55.6	0-32.7	
**History of initial treatment**			0.135
**Surgery only**	1	3	
**Surgery + radiochemotherapy**	18	11	
**Surgery + chemotherapy**	1	5	
**Surgery + radiotherapy**	2	3	

At the time of analysis, the median duration of follow-up was 31.2 weeks (range 1.1-238.4 weeks) for 44 patients. Main outcomes are listed in Table 2.

**Table 2:** Progression-free and overall survival

Comparison	Median Survival (weeks)	Minimum Survival (weeks)	Maximum Survival (weeks)	HR	95% CI	*P*
**Progression-free Survival**						
Overall ADV-TK *v* Control	29.6 *v* 8.4	7.9 *v* 1.1	238.4 *v* 39.1	0.315	0.161 to 0.615	0.001
GBM Subgroup ADV-TK *v* Control	34.9 *v* 7.4	9.0 *v* 1.1	238.4 *v* 35.3	0.157	0.062 to 0.398	<0.001
**Overall Survival**						
Overall ADV-TK *v* Control	45.4 *v* 14.3	7.9 *v* 1.1	238.4 *v* 45.6	0.207	0.096 to 0.444	<0.001
GBM Subgroup ADV-TK *v* Control	45.4 *v* 8.6	9.0 *v* 1.1	238.4 *v* 45.0	0.125	0.044 to 0.356	<0.001

Abbreviation: HR, hazard ratio; CI, confidence interval. HRs<1 indicate benefit with ADV-TK.

### PFS-6, PFS and OS in ADV-TK treated group and control group

**Figure 2:**
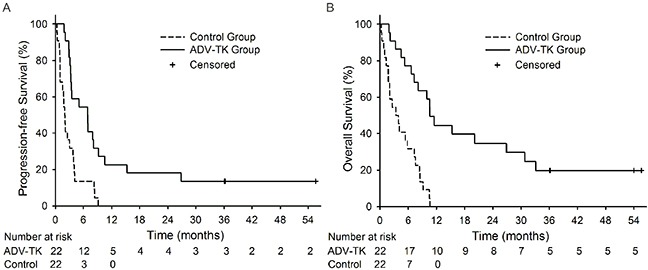
Kaplan-Meier plots for Progression-free Survival (PFS) and Overall Survival (OS) **A.** PFS for ADV-TK group versus Control group. **B.** OS for ADV-TK group versus Control group.

In ADV-TK treated group, the PFS-6 rate and median PFS were 54.5% and 29.6 weeks (range 7.9-238.4 weeks) respectively, much better than those in control group (PFS-6 = 13.6%; median PFS = 8.4 weeks, ranging 1.1-39.1 weeks). The actuarial probability of PFS rates was significantly different between two groups (HR: 0.315; 95% CI: 0.161 to 0.615; *P* = 0.001) (Figure 2A). At the end of the investigation, the median OS was 45.4 weeks (range 7.9-238.4 weeks) in ADV-TK treated group, more longer than that in control group (14.3 weeks, ranging 1.1-45.6 weeks). The difference in the OS rates between these two groups was significant (HR: 0.207; 95% CI: 0.096 to 0.444; *P* < 0.001) (Figure 2B). The one-year PFS rate and OS rate were 22.7% and 44.6% respectively in ADV-TK group. Among the 22 patients who received ADV-TK treatment, two patients remained alive until Oct 2014. In the control group, all patients died within one year. Adjustment by the Cox regression model did not substantially alter the results (Table 2).

### Objective response and overall clinical benefit in the ADV-TK group

All 22 patients in the ADV-TK group received objective-response evaluation at the end of the second treatment cycle. No complete response was observed in any patient. One patient was partial response (4.5%), 14 patients achieved stable disease (63.6%), and disease progression was observed in seven patients during treatment (31.8%). The clinical benefit (complete response, plus partial response, plus stable disease) of ADV-TK therapy was 68.2% at the end of the second cycle.

### PFS-6, PFS and OS for glioblastoma patients

**Figure 3:**
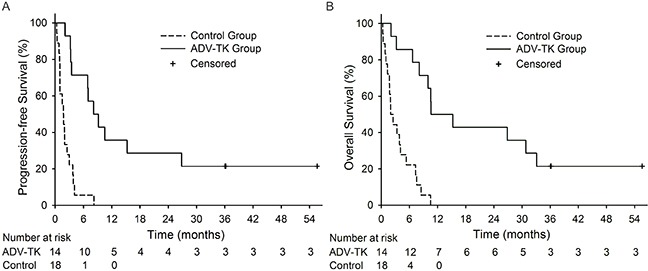
Kaplan-Meier plots for Progression-free Survival (PFS) and Overall Survival (OS) in GBM subgroup **A.** PFS for ADV-TK group versus Control group. **B.** OS for ADV-TK group versus Control group.

There were 32 patients with recurrent glioblastoma, 14 patients in the ADV-TK group and 18 patients in the control group. The PFS-6 rate was 71.4% in the ADV-TK group and 5.6% in the control group. Median PFS was 34.9 weeks (range 9.0-238.4 weeks) in the ADV-TK group and 7.4 weeks (range 1.1-35.3 weeks) in the control group respectively, showing the significant difference between these two groups (HR: 0.157; 95% CI: 0.062 to 0.398; *P* < 0.001) (Figure 3A). The median OS was 45.7 weeks (range 9.0-238.4 weeks) in the ADV-TK group and illustrated significant improvement when compared with control group (8.6 weeks, ranging 1.1-45.0 weeks) (HR: 0.125; 95% CI: 0.044 to 0.356; *P* < 0.001) (Figure 3B). The one-year PFS and OS rates in ADV-TK group were 35.7% and 50.0% respectively, with only one patient living till Oct 2014 (Table 2). All patients alive less than one year in the control group.

### Safety

ADV-TK treatment was well tolerated, and no treatment-related severe adverse events were observed. Mild catarrhal symptoms were reported in 5/22 patients who received ADV-TK therapy. Slight fever with no chills was also observed after injection of ADV-TK during the first three days in the same five patients (temperature range 37.3-38.3°C). Three of these five patients also suffered from light headache. All of these symptoms subsided within six days. One patient in the ADV-TK group experienced cerebral vasospasm 10 days after ADV-TK treatment. Hematological toxicity mainly consisted of grade 1 to 2 neutropenia, with only one patient in each group developing grade 3 neutropenia. There was no evidence of liver or kidney dysfunction caused by ADV-TK in our study with no significant difference between two groups. Toxicity experienced only during the first eight weeks of treatment was compared (Table 3).

**Table 3:** Toxicity of adv-tk in patients with malignant recurrent glioma

Toxicity	Grade (NCI-CTC)	ADV-TK group (*n*=22) No. of Patients (%)	Control group (*n*=22) No. of Patients (%)	*P*
**Catarrhal symptoms**		5 (22.7%)	-	-
**Anemia**		4 (18.2%)	6 (27.3%)	0.910
	I	2 (9.1%)	2 (9.1%)	
	II	1 (4.5%)	2 (9.1%)	
	III	1 (4.5%)	2 (9.1%)	
**Neutropenia**		6 (36.4%)	7 (31.8%)	0.878
	I	2 (9.1%)	3 (13.6%)	
	II	3 (13.7%)	3 (13.6%)	
	III	1 (4.5%)	1 (4.5%)	
**Thrombocytopenia**		4 (18.2%)	3 (13.6%)	0.915
	I	3 (13.2%)	2 (9.1%)	
	II	1 (4.5%)	1 (4.5%)	
**Nausea/vomiting**	I	7 (31.8%)	6 (27.3%)	0.815
**Cerebral vasospasm**	I	1 (4.5%)	0	-
**ALT**		3 (13.2%)	6 (27.3%)	0.396
	I	1 (4.5%)	5 (22.7%)	
	II	2 (9.1%)	1 (4.5%)	
**AST**	I	3 (13.2%)	5 (22.7%)	0.722

Abbreviation: NCI-CTC, National Cancer Institute Common Terminology Criteria for Adverse Events; ALT, alanine aminotransferase; AST, aspartate aminotransferase.

## DISCUSSION

Tumor-treatment strategies for recurrent HGGs remain imprecise. The lack of randomized studies comparing use of these agents with best supportive care may be an important limitation to determining the most effective tumor-treatment strategy. Recently, clinical trials demonstrating improved PFS-6 rates of up to 50% and response rates of up to 57% have resulted in accelerated United States Food and Drug Administration (FDA) approval of bevacizumab in patients with recurrent glioblastoma [1], but its use is associated with toxicities, eventual treatment resistance, and the potential for progression to a more invasive tumor phenol type. The unique combination of the high mitotic activity of tumor cells and the mostly post-mitotic environment of the adult brain, as well as aggression within the local environment within the closed compartment of the central nervous system [12], have been the subjects of many gene-therapy studies. Table 4 contains the most recent and clinically relevant approaches to virus-mediated gene therapy of recurrent HGGs. Although all of these clinical trials were phase I to II and included limited numbers of patients, their encouraging results highlight the general safety of the approach and their potential role in the treatment of recurrent HGGs.

**Table 4:** Clinical trials with gene therapy for recurrent HGG patients

Gene Therapy	Author (year)	Tumor Type	Phase	No. of Patients	PFS-6 (%)	Median PFS (months)	Median OS (months)
**AdHSV-TK/GCV**	Trask et al. (2000)^13^	Recurrent GBM	I	13	N/A	N/A	4.0 (post-GT) 20.9 (post-DX)
	Judy and Eck (2002)^14^	Primary and recurrent GBM	I	13	N/A	3	10.0
	Germano et al. (2003)^15^	Recurrent GBM	I	11	N/A	N/A	12.0 (post-GT) 22.0 (post-DX)
	Smitt et al. (2003)^16^	Recurrent GBM	I	14	N/A	2.3	4.0
	Immonen et al. (2004)^8^	Primary and recurrent GBM	II Randomized	36 (all) 17 (GT) 19 (Control)	N/A	N/A	62.4 weeks (GT) 55.3 weeks (GBM)
**Ad-p53**	Lang et al. (2003)^17^	Recurrent GBM	I	15	33.3% (5/15)	13 weeks	43 weeks
**ONYX-015**	Chiocca et al. (2004)^18^	Recurrent HGG	I	24	N/A	46 days	6.2 (all) 4.9 (GBM)
**G207**	Markert et al. (2000)^19^	Recurrent GBM	I	21	N/A	3.5 (all, mean)	12.8 (alive, mean) 6.2 (dead, mean) 15.9 (GBM, post-DX, mean)
	Market et al. (2009)^20^	Recurrent GBM	Ib	6	N/A	3.0	6.6 (post-GT) 23.0 (post-DX)
**HSV1716**	Rampling et al. (2000)^21^	Recurrent MG	I	9	N/A	N/A Range: 2.0 ∼ 24.0	N/A Range: 8 weeks ∼ 24 months
	Papanastassiou et al. (2002)^22^	Primary and recurrent GBM	I	12	N/A	N/A	greater than 7.0 Range: 1.0 ∼ 13.0
	Harrow et al. (2004)^23^	Primary and recurrent GBM	N/A	12	50% (6/12)	N/A	N/A Range: 3.0-22.0

Abbreviation: PFS, progression-free survival; PFS-6, 6-month progression-free survival; OS, overall survival; MG, malignant gliomas; GBM, glioblastoma; HGG, high grade glioma; DX, diagnosis; GT, gene therapy; N/A, data not available.

Our phase II, randomized clinical trial demonstrated clear progression-free survival and overall survival benefit associated with ADV-TK gene therapy, which was also shown to be safe. Even though our trial includes a small number of patients, to our knowledge this patient group is the largest of those included in adenovirus gene-therapy clinical trials with recurrent HGGs.

The results of our current investigation present an advantage in the strategy of administration of ADV-TK gene reagent. In order to disrupt the blood-brain barrier, mannitol was administrated just before every ADV-TK/ganciclovir intra-arterial infusion. Mannitol has been used as regular clinical treatment for intracranial hypertension for many years [24, 25]. As early as 1972, Raport et al proposed the theory of application of hypertonic solution to open the blood-brain barrier [26]. Later, Neuwelt et al applied the theory in the intra-arterial chemotherapy in brain tumor [27]. In our study, ADV-TK/ganciclovir intra-arterial infusion brought it's superiority into full efficiency by assist of mannitol administration. The values of PFS-6 rate, median PFS and median OS in our study were significantly higher than those among the relevant virus-mediated gene therapy clinical trials (Table 4), and also better than those of the clinical trials with chemotherapy and NovoTTF-100A (Supplementary Table).

We previously reported two clinical trials supporting the efficacy and safety of ADV-TK gene therapy [10, 11]. The adverse effects in present investigation were consistent with our previous studies. Fever was one of the most common treatment-related adverse events and may be due to a physical immune response to the vector. Another adverse effect was hematological toxicity which mainly consisted of grade 1 to 2 neutropenia. There were three patients had light headache after treatment which was supposed to the physiological reaction of mannitol administration. Fortunately, all of these responses were transient and easy to tolerate.

The number of patients enrolled in the current clinical trials is the largest patient population among the virus-mediated gene therapy trials in Table 4, but is still small size of clinical trials. The next we need to do is to increase the number of patients and compare with a single agent or a combination regimen containing TMZ, bevacizumab, or irinotecan. Concerning local gene therapy, the greatest shortcoming of non-replicating adenovirus gene therapy appears to be the low transduction efficiency of the vector and its limited distribution. Recently, our research group developed a series of conditionally replicating virus vectors [28–30], by which therapeutic gene should express more effectively in target tumor cells. It will be a good choice for further studies of ADV-TK gene therapy in the treatment of malignant tumors.

## MATERIALS AND METHODS

This study was conducted in accordance with the Declaration of Helsinki. All participating institutions obtained local ethics approval and written informed consent from all patients. This trial is registered with ClinicalTrials.gov. NCT00870181.

### Patients

From November 2009 to December 2012, patients were enrolled at Beijing Tiantan Hospital, Beijing Chao-Yang Hospital, and Beijing Friendship Hospital. Eligible patients were adults of either sex with histologically confirmed WHO grades 3 to 4 malignant glioma (glioblastoma multiforme, anaplastic astrocytoma, or anaplastic oligodendroglioma) who had just received diagnoses of recurrence or progression based on clinical or radiological evidence upon presentation to the centers. Patients were fit for intra-arterial infusion and intravenous chemotherapy, with adequate hepatic, renal, and hematologic function. Evidence of recurrence or progression was confirmed by evaluable enhancing recurrent tumor on contrast-enhanced magnetic resonance imaging before and after the treatment. Patients with primary WHO grade 1 to 2 glioma were pathologically verified with disease recurrence or progression to WHO grade 3 to 4. Additional eligibility criteria included legal age ≥18 years, an Eastern Cooperative Oncology Group performance ≥2, chemotherapy completion ≥4 weeks prior, and recovery from drug-induced toxicities. The exclusion criteria were active pregnancy, prior gene therapy, second primary tumor, gravidity, lactation, hypersensitivity to antiviral drugs, immunological deficit, active uncontrolled infections, or requiring treatment with warfarin or any other anticoagulants.

### Treatment

Patients were randomly allocated to either the ADV-TK group or to the control group according to the random table kept in Tiantan Hospital affiliated with Capital Medical University (Beijing, P.R. China). ADV-TK is a chimeric human group C adenovirus (ADV5) that expresses the gene encoding HSV-TK under the control of a Rous sarcoma virus long terminal repeat promoter, which is inserted in the region of the excised E1 adenoviral genes. In the ADV-TK group, ADV-TK (Tian Dakang Co.) was administered via intra-arterial cerebral infusion. Based on the toxicity results from our previous study, a total of 1 × 10^12^ viral particles of ADV-TK was administered in the current clinical trial [10].

Systemic ganciclovir therapy was delivered at a dose of 5 mg/kg intravenous, every 12 h at 36 h after ADV-TK therapy for 14 days. Mannitol (25% 1.4 M mannitol, 250 ml/15 minutes) was given just before every ADV-TK /ganciclovir infusion to disrupt the blood-brain barrier. The ADV-TK/ganciclovir treatment was repeated every 21 days for at least two cycles. Patients in the control treatment group received surgery or systemic chemotherapy or palliative care. Imaging (using the same contrast-enhanced imaging method as the baseline scan) was repeated on clinical progression wherever possible; scans were also performed at baseline and at the end the second and fourth cycles for the ADV-TK group, with progressive disease defined as a ≥25% increase in two-dimensional tumor size. Safety was assessed according to National Cancer Institute Common Terminology Criteria for Adverse Events (Version 3.0).

### Study measures and statistical analysis

The primary end point was PFS-6. Secondary end points included PFS, OS, safety, objective response rate (complete response, partial response, stable disease, and progressive disease), and clinical benefit (complete response, plus partial response, plus stable disease). PFS and OS were calculated from the day of randomization until the event (progression confirmed either radiologically or clinically if scan not performed or death) happened or until the patient was censored. Event-free patients were censored on the date of last follow-up.

Kaplan-Meier curves were compared using the log-rank test. Treatment hazard ratios (HRs) with a 95% confidence interval (CI) were calculated using the Cox regression model. For comparisons against the control group, HR < 1 indicate benefit with ADV-TK. Hypothesis testing was two-sided. *P* values < 0.05 were considered to indicate statistical significance. Data were analyzed with SPSS statistical software (version 18·0 for Windows, SPSS).

## SUPPLEMENTARY TABLE


