# Supplementary material for: Adenovirus-mediated delivery of herpes simplex virus thymidine kinase administration improves outcome of recurrent high-grade glioma
Source: Oncotarget. 2015 Dec 23;7(4):4369–78. doi: 10.18632/oncotarget.6737 (PMC4826211; doi:10.18632/oncotarget.6737)
Supplement: Supplementary file 1 [file oncotarget-07-4369-s001.pdf]

SUPPLEMENTARY TABLE

Supplementary Table S1: Treatments for recurrent HGG

Treatment	Author (year)	Tumor Type	PFS-6 rate (%)	Median PFS (months)	Median OS (months)	Clinical Benefit (%) (CR+PR+SD)
**TMZ**	Perry et al. (2008)[17]	Recurrent GBM	17% (group 1, GBM)<br>57% (group 2, GBM)	N/A	N/A	47.3% (group 1)<br>78.6% (group 2)
	Perry et al. (2010)[30]	GBM with progression	23.9% (all)<br>27.3% (early)<br>7.4% (extended)<br>35.7% (rechallenge)	N/A<br>3.6 (early)<br>1.8 (extended)<br>3.7 (rechallenge)	N/A	N/A<br>27.3% (early)<br>7.7% (extended)<br>37.0% (rechallenge)
**Bevacizumab (BV)**						
BV	Kreisl et al. (2009)[31]	Recurrent GBM	29%	16 weeks	31 weeks	35% (CR+PR)
BV + CPT	Vredenburgh et al. (2007)[32]	Recurrent GBM	46%	24 weeks	42 weeks	57% (CR+PR)
	Gil et al. (2012)[33]	Recurrent MG	N/A<br>46% (GBM)	5.1 (all)<br>5.1 (GBM)	9 (all)<br>8.8 (GBM)	71% (all)<br>67.4% (GBM)
BV / BV + CPT	Friedman et al. (2009)[18]	Recurrent GBM in first or second relapse	42.6% (BV)<br>50.3% (BV+CPT)	4.2 (BV)<br>5.6 (BV+CPT)	9.2 (BV)<br>8.7 (BV+CPT)	28.2% (BV)<br>27.2% (BV+CPT)<br>(CR+PR)
BV + CT	Norden et al. (2008)[34]	Recurrent MG	39%<br>42% (GBM)	23.5 weeks	35.7 weeks	93.2% (CR+PR+MR+SD)
BV + Nitrosourea	Soffietti et al. (2009)[35]	Recurrent MG	N/A	N/A	N/A	35% (CR+PR)
BV + TMZ	Verhoeff et al. (2010)[36]	Recurrent HGG	17.4% (all)<br>6.7% (GBM)	13.9 weeks (all)<br>10.4 weeks (GBM)	17.1 weeks (all)<br>15.7 weeks (GBM)	20% (all)<br>16.7% (GBM)<br>(CR+PR)
**Nitrosourea**						
Lomustine	Wick et al. (2010)[37]	Recurrent GBM	19.0%	1.6	7.1	40.2%
Fotemustine (FTM)	Paccapelo et al. (2012)[38]	Recurrent or progressive GBM	33.1% (All)<br>24% (early)<br>28.9% (extended)<br>31.3% (rechallenge)	N/A	N/A	47.9% (All)<br>45% (early)<br>36% (extended)<br>42.2% (rechallenge)
PCV	Brada et al. (2010)[39]	Recurrent HGG	N/A	3.6	6.7	-
**NovoTTF-100A**	Stupp et al. (2012)[40]	Recurrent GBM	21.4%	2.2	6.6	14% (CR+PR)

Abbreviation: PFS, progression-free survival; PFS-6, 6-month progression-free survival; OS, overall survival; CR, complete response; PR, partial response; SD, stable disease; MR, minimal response; MG, malignant gliomas; GBM, glioblastoma; HGG, high grade glioma; TMZ, temozolomide; BV, bevacizumab; CT, chemotherapy; FTM, fotemustine; TTF, tumour treating fields; PCV, procarbazine + lomustine + vincristine; N/A, data not available.
